# Clinical pharmacogenomic testing of *KRAS, BRAF *and *EGFR *mutations by high resolution melting analysis and ultra-deep pyrosequencing

**DOI:** 10.1186/1471-2407-11-406

**Published:** 2011-09-24

**Authors:** Emma Borràs, Ismael Jurado, Imma Hernan, María José Gamundi, Miguel Dias, Isabel Martí, Begoña Mañé, Àngels Arcusa, José AG Agúndez, Miguel Blanca, Miguel Carballo

**Affiliations:** 1 Molecular Genetics Unit, Hospital de Terrassa, Ctra. Torrebonica, 08227 Terrassa, Spain; 2 Pathology Service, Hospital de Terrassa, Ctra. Torrebonica, 08227 Terrassa, Spain; 3 Oncology Service, Hospital de Terrassa, Ctra. Torrebonica, 08227 Terrassa, Spain; 4 Department of Pharmacology, Universidad de Extremadura, Av. Elvas, 06071 Badajoz, Spain; 5 Allergy Service, Hospital Carlos Haya, Pl. Hospital Civil, 29009 Málaga, Spain

## Abstract

**Background:**

Epidermal growth factor receptor (*EGFR*) and its downstream factors *KRAS *and *BRAF *are mutated in several types of cancer, affecting the clinical response to EGFR inhibitors. Mutations in the *EGFR *kinase domain predict sensitivity to the tyrosine kinase inhibitors gefitinib and erlotinib in lung adenocarcinoma, while activating point mutations in *KRAS *and *BRAF *confer resistance to the anti-EGFR monoclonal antibody cetuximab in colorectal cancer. The development of new generation methods for systematic mutation screening of these genes will allow more appropriate therapeutic choices.

**Methods:**

We describe a high resolution melting (HRM) assay for mutation detection in *EGFR *exons 19-21, *KRAS *codon 12/13 and *BRAF *V600 using formalin-fixed paraffin-embedded samples. Somatic variation of *KRAS *exon 2 was also analysed by massively parallel pyrosequencing of amplicons with the GS Junior 454 platform.

**Results:**

We tested 120 routine diagnostic specimens from patients with colorectal or lung cancer. Mutations in *KRAS*, *BRAF *and *EGFR *were observed in 41.9%, 13.0% and 11.1% of the overall samples, respectively, being mutually exclusive. For *KRAS*, six types of substitutions were detected (17 G12D, 9 G13D, 7 G12C, 2 G12A, 2 G12V, 2 G12S), while V600E accounted for all the *BRAF *activating mutations. Regarding *EGFR*, two cases showed exon 19 deletions (delE746-A750 and delE746-T751insA) and another two substitutions in exon 21 (one showed L858R with the resistance mutation T590M in exon 20, and the other had P848L mutation). Consistent with earlier reports, our results show that *KRAS *and *BRAF *mutation frequencies in colorectal cancer were 44.3% and 13.0%, respectively, while *EGFR *mutations were detected in 11.1% of the lung cancer specimens. Ultra-deep amplicon pyrosequencing successfully validated the HRM results and allowed detection and quantitation of *KRAS *somatic mutations.

**Conclusions:**

HRM is a rapid and sensitive method for moderate-throughput cost-effective screening of oncogene mutations in clinical samples. Rather than Sanger sequence validation, next-generation sequencing technology results in more accurate quantitative results in somatic variation and can be achieved at a higher throughput scale.

## Background

The epidermal growth factor receptor (EGFR) plays a key role as a receptor tyrosine kinase (TK), controlling several signalling pathways that stimulate cell growth, proliferation and survival. Mutations involving the EGFR axis can cause its constant activation, leading to uncontrolled cell proliferation. Not surprisingly, *EGFR *mutations have been identified in several types of cancer and it is a target of many anticancer therapies, including small-molecule TK inhibitors (e.g., gefitinib and erlotinib for lung cancer) and monoclonal antibodies (e.g., cetuximab and panitumumab for colon cancer). Moreover, the mutational status of EGFR and its downstream molecules have implications for the responsiveness to treatment and prognosis.

Somatic mutations in the kinase domain of the *EGFR *gene (exons 18-21) are reportedly associated with sensitivity of lung cancers to TK inhibitors [1-5]. About 90% of the sensitising mutations are in-frame deletions in exon 19, affecting the conserved amino acids LREA, and the point mutation L858R in exon 21. Such *EGFR *mutations increase sensitivity to TK inhibitors, most likely through induction of critical structural modifications of the ATP-binding site in the TK domain. Unfortunately, during the course of treatment, some patients eventually develop acquired resistance to TK inhibitors, often due to the secondary T790M mutation in *EGFR *exon 20 [6,7]. Furthermore, a significant proportion of cancer patients show no benefit from anti-EGFR therapies because of the independent activation of downstream signalling, especially the Ras/Raf/MAPK pathway. Mutations in the *KRAS *gene occur early in the development of many cancers and are found in more than 90% of pancreatic adenocarcinomas, 40% of colorectal cancers (CRC) and 33% of non-small cell lung carcinomas (NSCLC) [8]. Commonly restricted to codon 12/13 in exon 2, and rarely codons 59 and 61 in exon 3 [8,9], these mutations cause impaired GTPase activity and result in a continual stimulus for cellular proliferation. Somatic *KRAS *mutations have been associated with resistance to EGFR-targeted agents in lung cancer and metastatic CRC [10], and are mutually exclusive with *EGFR *mutations in large series of NSCLC [4,11].

Likewise, *KRAS *and *BRAF *mutations are inversely associated in CRC, consistent with the fact that both induce similar effects through the same pathway, since the B-Raf protein kinase is activated by membrane-bound Ras. *BRAF *mutations are found in many types of cancer, predominantly in up to 80% of melanoma and nevi [12]. V600E amino acid substitution in the activation segment accounts for 90% of *BRAF *mutations and is significantly associated with microsatellite instability [13]. Data from retrospective studies suggest that mutated *BRAF*, which is present in 5-10% of colorectal tumours, can affect the response to anti-EGFR monoclonal antibodies in patients with wild type *KRAS *[14-16], 40-60% of whom do not respond to such therapy [17].

Current guidelines in the US state that patients with metastatic CRC being considered for EGFR-targeted therapies should be tested for *KRAS *and *BRAF *mutations [18], and recommend *EGFR *testing for patients with advanced NSCLC to predict response to first-line TK inhibitors [19,20]. Moreover, the European Society of Pathology has started a helpdesk and a quality assurance program for *KRAS *testing in CRC [21]. Rapid, sensitive and reliable methods for mutation detection are therefore required for stratification of patients to receive molecularly targeted treatment.

High-resolution melting (HRM) is a recently developed technique that shows great potential for scanning germline and somatic mutations [22]. This method is based on a real-time PCR amplification in the presence of a saturating intercalating fluorescent dye and subsequent separation of the DNA strands in a temperature gradient, during which the fluorescence is registered with high resolution. If there are mutant alleles in the sample, the formed heteroduplexes of wild type and mutant alleles are separated at lower temperatures, generating a different melting pattern. The HRM assay is useful as a pre-screening test and positive samples should be sequenced to identify the specific nucleotide alterations. However, at the limit of somatic mutation detection with real time PCR technologies, sequencing by Sanger is not suitable as a confirmatory method and validation of results may require a more quantitative sequence variation assay.

Ultra-deep sequencing is undoubtedly the most sensitive technology currently available for mutation scanning. This method is well suited for detection of somatic mutations, which may be present in a small fraction of tumour cells within a background of normal tissue. Benchtop sequencers, like the GS Junior 454, have brought high-throughput sequencing to molecular diagnostic laboratories, and development of cancer mutation detection assays using this platform is therefore of special interest.

Herein, we describe a HRM assay to identify hotspot mutations in *EGFR*, *KRAS *and *BRAF *oncogenes, and investigate the potential application of ultra-deep amplicon pyrosequencing for somatic variation detection in clinical samples.

## Methods

### Patient samples

Informed consent was obtained from all patients prior to the study, which was conducted in accordance with the Helsinki Declaration and approved by the internal Clinical Research Ethics Committee (CEIC) of the Hospital de Terrassa (Spain). Analysis of *KRAS*, *BRAF *and *EGFR *mutations was performed in 120 formalin-fixed paraffin-embedded (FFPE) tumour samples, according to the clinicians' orders. The specimens included 81 CRCs, 27 lung carcinomas and 12 metastatic tumours from primary colorectal (n = 2) or lung (n = 10) cancers. Before starting the routine mutation testing, an additional series of 32 CRC samples was used to compare our results with those obtained in another centre using the DxS TheraScreen *KRAS *mutation kit (Qiagen, Izasa, Barcelona, Spain). For *KRAS *mutation screening by ultra-deep pyrosequencing, five known CRC samples were selected, including two wild type (33K and 75K) and three mutants: 51K (G12V), 81K (G12D apparently heterozygous) and 97K (G12C).

### DNA extraction

Tumour-rich areas marked by the pathologist on a hematoxylin and eosin histologic section were manually macrodissected by block trimming or target tissue dissection, and up to 10 sections of 5 μm thickness were collected in a microtube for genetic testing. In general, the estimated percentage of cancer cells in the selected tissue was greater than 70% of total cells. Genomic DNA was extracted using the QIAamp DNA FFPE Tissue Kit (Qiagen), quantitated with the Epoch Multi-Volume Spectrophotometer System (BioTek, Izasa, Barcelona, Spain), and stored at -20°C until use.

### Mutation screening by HRM

#### Design of HRM primers

At first, existing primer pairs from previously published HRM studies were tested to ensure good discrimination between wild type and mutant samples in our platform. If they were inappropriate or unavailable, new primers were designed. Since FFPE-derived DNA is often fragmented and because the influence of variation in the melting curve shape decreases with an increasing sequence length, short amplicons were preferred. The inclusion of single nucleotide polymorphisms (SNPs) within the amplicon was minimised and, if the primer was placed over a sequence variant, mismatched bases with no allelic preference were introduced. Primer pairs for all target regions were analysed for specificity and to ensure similar melting temperatures using Primer-BLAST software [23], and DNA melting predictions were performed with the web servers Stitchprofiles.uio.no [24] and DINAMelt [25]. As shown in Table 1, existing primers were used for *KRAS *[26] and *EGFR *exons 19 and 21 [27], and newly designed primers for *EGFR *exon 20 and *BRAF*. All the amplicons spanned ≤250 bp and covered most common mutations.

**Table 1:** HRM primer sequences

Gene	Exon	Primer sequence	Amplicon size
***KRAS***	**2**	F: 5'-GGCCTGCTGAAAATGACTGAATATAA-3'	170 bp [26]
		R: 5'-AAAGAATGGTCCTGCACCAGTA-3'	

***BRAF***	**15**	F: 5'-TCATGAAGACCTCACAGTAAAAATAGG-3'	164 bp
		R: 5'-AGCAGCATCTCAGGGCCAAA-3'	

***EGFR***	**19**	F: 5'-GTGCATCGCTGGTAACATCCA-3'	250 bp§ [27]
		R: 5'-AAAGGTGGGCCTGAGGTTCA-3'	

***EGFR***	**20**	F: 5'-ACCTCCACCGTGCA(T*)CTCAT-3'	128 bp
		R: 5'- ATTACCTTTGCGATCTGAACACACC -3'	

***EGFR***	**21**	F: 5'-CCTCACAGCAGGGTCTTCTCTG-3'	210 bp§ [27]
		R: 5'-TGGCTGACCTAAAGCCACCTC-3'	

F: forward, R: reverse.

§ These amplicons span the entire exon.

(*) Mismatched base introduced to prevent SNP interference (c.2361G > A) and allelic preference.

#### HRM assay

Samples were assayed in duplicate using the LightCycler 480 system (Roche, Barcelona, Spain). Each 10 μl reaction contained about 70 ng DNA diluted in 1.8 μl, 1x LightCycler HRM Master Reaction Mix (Roche), 3 mM MgCl_2_, and 200 nM primers (HPLC purified). Wild type and non-template control samples were added for each amplicon tested. The same touchdown PCR program and melting conditions were used for all amplicons: 95°C for 10 min; 45 cycles of 95°C for 10 s, 62-54°C (1°C/cycle) for 15 s and 72°C for 10 s; 95°C for 1 min; 40°C for 1 min; a melt of 72-92°C (0.01°C/s, 45 acquisitions/°C); and 40°C for 10 s. Data were acquired and analysed with the accompanying Gene Scanning software. Normalised and temperature-adjusted melting curves of test samples and wild type controls were compared, and samples with an aberrant melting pattern were judged to carry a somatic mutation.

#### Sanger sequencing

Amplified products of mutation positive or ambiguous samples were recovered from the plate, column purified with the High Pure PCR Product Purification Kit (Roche), and submitted to StabVida (Oeiras, Portugal) for direct sequencing on a 3730XL ABI DNA sequencer (Applied Biosystems, Foster City, CA) using the Big Dye terminator V1.1 DNA sequencing kit and the HRM primers.

#### HRM sensitivity testing

To test the analytical sensitivity of our HRM assay we used patient FFPE-derived DNA, assuming that a sample carrying a heterozygous (Ht) mutation contained 50% mutant alleles. Genomic DNA of the *KRAS *mutant sample 81K (G12D Ht) was mixed with wild type DNA of sample 75K in dilutions containing 50%, 25%, 10%, 5%, 2.5% and 0% mutant alleles. Moreover, decreasing amounts of template DNA were used to investigate the limits of *KRAS *mutation detection. Samples 81K (G12D Ht) and 100K (wild type) were compared using 40, 20, 10 and 5 ng DNA.

### Mutation screening by ultra-deep pyrosequencing

#### Design of fusion primers

**Figure 1:**
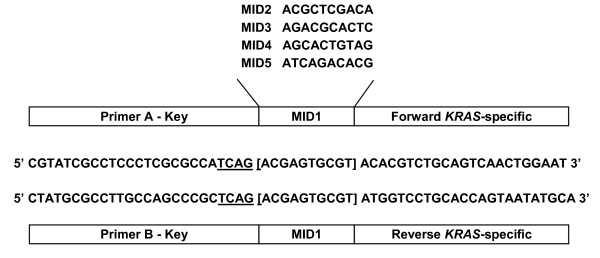
**Fusion primer design for *KRAS *screening with the GS Junior 454 system**. The primers consist of three parts: the GS Junior 454 adaptor, containing the sequencing primers A and B and the sequencing key "TCAG" (underlined), followed by a 10-bp multiplex identifier (MID) tag that varies between samples, and the *KRAS*-specific sequence. Five primer pairs were designed using MIDs 1 to 5 and generating an amplicon of 409 bp that contains *KRAS *exon 2. Complete sequences of forward and reverse primers are shown for MID1.

The primers used to generate *KRAS *amplicon libraries were composed of three parts fused together (Figure 1). The 5'-portion was a 25-mer corresponding to the adaptors A and B required by the 454 sequencing system and ending with the sequencing key "TCAG". Multiplex Identifiers of 10 bp (MIDs 1-5 from the standard 454 set), placed after the key, were used to barcode the different samples. And the 3'-portion was designed to anneal with a specific sequence on either side of *KRAS *exon 2, resulting in a 409 bp amplicon (total size with adaptors and MIDs). The presence of the same MID tag in the forward and reverse primer of each pair, and the length of the generated amplicons, within the 400-500 bp range offered by Titanium chemistry, allowed for bidirectional sequencing with primers A and B.

#### Amplicon preparation

PCR was conducted in a 50 μl final volume containing: 1x PCR buffer, 1.5 mM MgCl_2_, 500 nM fusion primers, 2 μl genomic DNA (0.3-1.5 μg), 200 μM dNTPs, 2.5 U of BioTaq polymerase (Bioline, Ecogen, Barcelona, Spain) and PCR grade water. Program conditions were: 5 min at 95°C; followed by 35 cycles of 30 s min at 95°C, 30 s at 59°C and 30 s at 72°C; and 8 min at 72°C. PCR products were analysed by 1.5% agarose gel electrophoresis and column purified with the High Pure PCR Purification Kit (Roche). Amplicons were further purified with Agencourt AMPure XP beads (Beckman Coulter, Izasa, Barcelona, Spain), and quantitated by fluorometry in a LightCycler 480 instrument (Roche) using the Quant-iT PicoGreen dsDNA Assay Kit (Invitrogen), as described by the manufacturer. The five amplicon libraries, with concentrations ranging from 6.63 × 10^9 ^to 5.05 × 10^10 ^molecules/μl, were equimolarly pooled to create a *KRAS *multiplexed library at 1 × 10^7 ^molecules/μl.

#### Pyrosequencing

To make the most of the capacity of the sequencing plate, this library was mixed with another of *BRCA *amplicons to be sequenced in a single run. Emulsion PCR of the combined library was carried out using the GS Junior Titanium emPCR Kit (Lib-A) and pyrosequenced on the GS Junior 454 platform (Roche). An input of 3 molecules of library DNA per capture bead was used and 500,000 enriched beads were loaded on the instrument. The library was sequenced in a Titanium PicoTiterPlate (PTP) with Titanium reagents, and base calling was performed with the amplicon filter settings.

#### Data analysis

Processed and quality-filtered reads were analysed with the GS Amplicon Variant Analyzer. The *KRAS *amplicon (excluding adaptors and MIDs) was used as the reference to align amplicon reads, template-specific portions of the fusion primers were considered as the forward and reverse primer, and the known mutations of the samples selected were defined as substitutions relative to the reference sequence. Correspondence of samples and MID tags was specified and, as the same MID was present in both orientations, an "either" encoding multiplexer was used to demultiplex the reads.

## Results

### Validation and sensitivity of *KRAS *HRM assay

**Figure 2:**
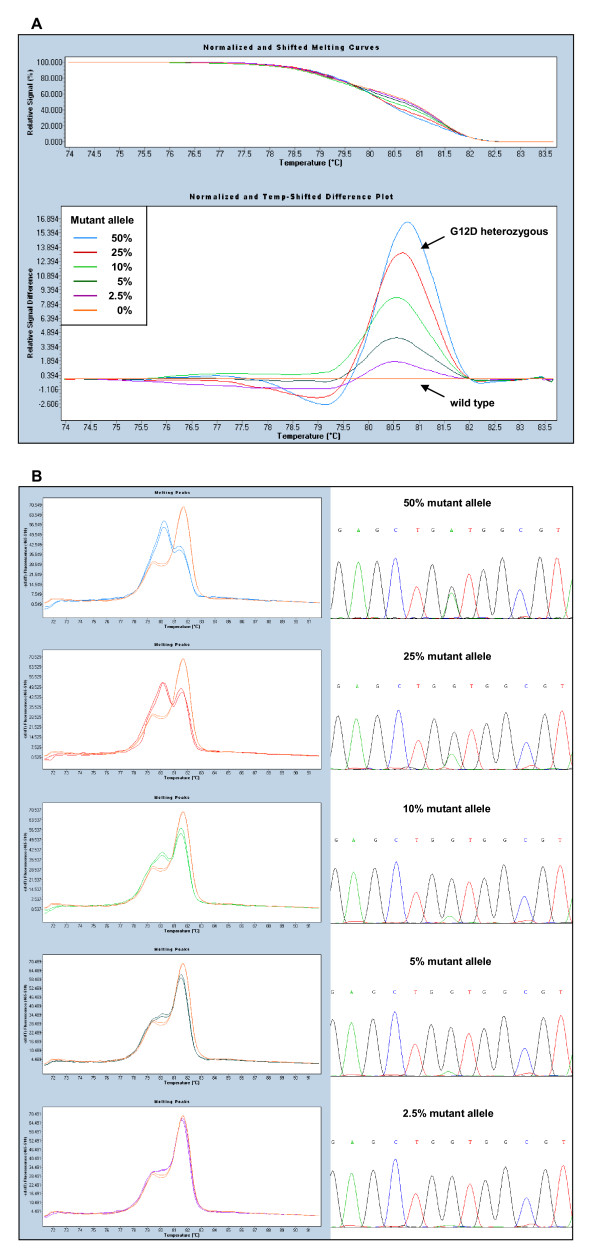
**Sensitivity of the *KRAS *HRM assay**. Mixtures of mutant (G12D heterozygous) and wild type genomic DNA samples reveal gradual curves. A) Adjusted melting curves (top) and differential plots (bottom) showing the presence of 50%, 25%, 10%, 5%, 2.5% and 0% mutant alleles. B) Melting peaks of mutant sample dilutions compared to the wild type control (left) and corresponding sequence traces (right). In the chromatograms, mutation peaks can be distinguished from the background because they are symmetrical and vertically aligned with the wild type peaks.

Mixtures of *KRAS *mutant (G12D Ht) and wild type DNAs were used to test the analytical sensitivity of the HRM assay. Figure 2 shows the difference plots (A) and melting peaks (B) for different dilutions of mutant DNA, and demonstrates that this assay can detect 2.5% mutant alleles, which corresponds to 5% tumour cells carrying a heterozygous mutation.

Wild type *KRAS *amplicons have a biphasic melting curve due to the presence of different melting domains (Figure 2-A). Accordingly, two melting peaks are observed in the first-derivative plot of normal samples, while mutant samples show another peak, generated by heteroduplexes melting, the height of which is proportional to the fraction of cells bearing the mutation (Figure 2-B). This peak alters considerably the characteristic shape of the curve, even with low amounts of mutant DNA. Thereby, the presence of 2.5% mutant alleles is visible in the graph but not in the chromatogram (Figure 2-B), where the peaks can be confused with the background, consistent with the higher sensitivity of HRM compared to direct Sanger sequencing.

On the other hand, the limited amount of FFPE tissue is a common problem, especially when working with lung cancer specimens. To investigate the lower limit of *KRAS *mutation detection, the HRM assay was performed with template DNA amounts of G12D Ht and wild type samples ranging from 40 to 5 ng. Mutant samples showed an abnormal profile across the entire range of template amounts tested and could be readily identified (not shown). Therefore, as little as 5 ng (or even less) of mutant DNA would be enough to detect the *KRAS *mutation in non-degraded samples.

### *KRAS*, *BRAF *and *EGFR *mutation detection by HRM

**Figure 3:**
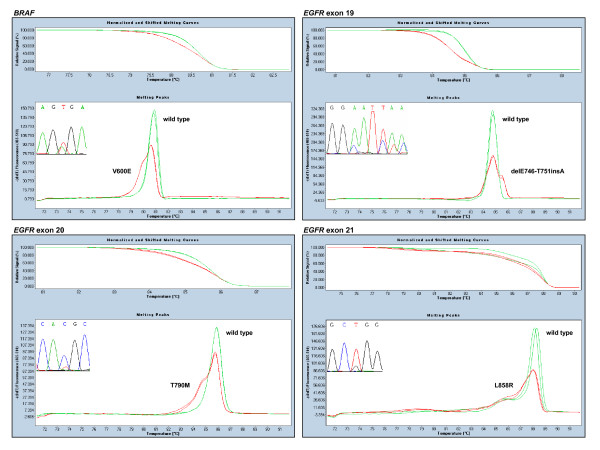
**Panel of HRM assays and sequence traces for *BRAF *and *EGFR***. Normalised shifted melting curves and melting peaks of HRM amplicons are compared between wild type (green) and representative mutant samples (red). Mutant samples show left-shifted curves and aberrant melting profiles, with a lower homoduplex peak and a more or less visible heteroduplex peak on its left, except for *EGFR *exon 19 deletions, in which the sequence of the deleted allele has a higher melting temperature.

A total of 120 routine diagnostic FFPE samples from patients with colorectal (n = 81), lung (n = 27) and other (n = 12) cancers were screened for mutations in the *EGFR *pathway. Melting patterns and sequence traces of representative mutations for each amplicon are depicted in Figure 2-B and Figure 3, with Table 2 showing the results obtained.

**Table 2:** Variants detected by HRM analysis

Gene	Nucleotide	Protein	No. detected	Total %
***KRAS***	35G > A	G12D	17	18.3
	38G > A	G13D	9	9.7
	34G > T	G12C	7	7.5
	35G > C	G12A	2	2.2
	35G > T	G12V	2	2.2
	34G > A	G12S	2	2.2
	Total no. of cases with mutated *KRAS*		**39**	**41.9**
	Total no. of cases		**93**	100

***BRAF***	1799T > A	V600E	7	13.0
	Total no. of cases with mutated *BRAF*		**7**	**13.0**
	Total no. of cases		**54**	100

***EGFR***	2236-2250del15	delE746-A750	1	2.8
	2237-2252del16	delE746-T751insA	1	2.8
	2369C > T, 2573T > G	T790M, L858R	1	2.8
	2543C > T	P848L	1	2.8
	Total no. cases with mutated *EGFR*		**4**	**11.1**
	Total no. of cases*		**36**	100

* Exon 20 was not analysed in 13 samples.

For *KRAS*, six different substitutions were detected at codon 12/13, predominantly G12D, whereas V600E accounted for all the *BRAF *mutations found (Table 2). Five mutations in the *EGFR *gene were detected in four patients. Two patients had exon 19 deletions (delE746-A750 and delE746-T751insA), one patient showed the common sensitising mutation L858R in exon 21 together with the resistance mutation T590M in exon 20, and the other harboured the uncommon exon 21 point mutation P848L, which appears to behave as a functionally silent polymorphism [28] (Table 2). In addition, a SNP in exon 21 (2508C > T at R836) was also detected in one patient.

All the mutant samples identified by HRM were confirmed by direct Sanger genomic sequencing. In addition, 28 random samples identified as wild type in the HRM analysis, as well as three samples with ambiguous melting patterns due to poor amplification, showed wild type chromatograms. Therefore, using HRM and Sanger sequencing, no false positives were detected in the 51 mutations assessed and no false negatives were found among the 31 cases analysed.

Only three specimens that showed insufficient amplification in the HRM assay because of the low amount or quality of the starting material were subjected to standard PCR amplification using the HRM primers followed by direct sequencing. Minimisation of reaction-to-reaction variability was especially important in these cases. To standardise sample preparation, we quantitated the extracted DNA by spectrophotometry, adjusted the samples to the same concentration and used the same amount of template in each reaction. Even though 5 ng is allowed for proper amplification of good quality samples, an excess of template (about 70 ng) was used to overcome the challenge posed by compromised DNA quality. Previous reports consistently suggest that inclusion of >30 ng purified DNA increases the success rate up to 96% [29]. For good HRM analysis, amplification curves were checked to produce a crossing point < 30 and to reach a similar plateau height. If duplicates of a sample showed different melting patterns, the assay was repeated for that sample. And when insufficient amplification precluded detection of subtle differences between small melting peaks, PCR products were sequenced to avoid false negative results.

### *KRAS *mutation detection by ultra-deep pyrosequencing

A pilot experiment was set up to evaluate the feasibility of applying amplicon pyrosequencing to detect somatic variation in FFPE tumour samples. With this aim, two wild type and three known mutant samples were selected for screening the entire *KRAS *exon 2.

**Figure 4:**
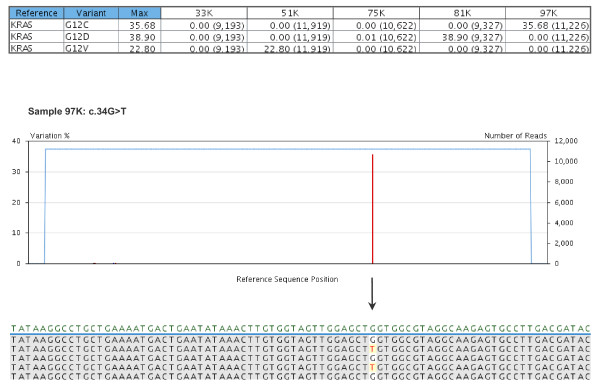
**Variants detected by ultra-deep pyrosequencing of *KRAS *amplicons**. The variants frequency table (top) summarises the frequencies of the previously defined variants detected within each sample, with the corresponding number of combined reads shown between parentheses. Below, the plot of sequence variations detected in sample 97K and a partial image capture of the global alignment, displayed as consensus reads, are shown as an example of 454 sequencing output. The only change detected within this sample is the known c.34G > T mutation (G12C).

A multiplexed *KRAS *amplicon library was prepared, mixed with *BRCA *amplicons, and subsequently sequenced with GS Junior 454, achieving a throughput of 26 million high-quality filtered bases and 7.7 × 10^4 ^filtered reads (41.3% key pass reads) for the library (containing the "TCAG" key). Considering only the *KRAS *amplicons, 9 to 12 thousand reads were obtained for each of the five samples selected (Figure 4).

The known *KRAS *genotype was validated in all cases, and frequencies of the previously defined variants were calculated from combined data of forward and reverse reads. Samples 33K and 75K were confirmed as wild type, while mutant samples showed the following variation frequencies: 22.8% G12V for 51K, 35.7% G12C for 97K, and 38.9% G12D for 81K. No additional variants were detected above the minimum frequency setting of 1% even though a DNA polymerase enzyme without proofreading activity was used in the PCR amplification. An illustrative example of the sequence variations detected within sample 97K is shown in Figure 4.

In the case of sample 81K, considered heterozygous on the basis of the Sanger chromatogram (Figure 2-B, top right), the observed percentage of 38.9% mutant alleles is in accordance with the estimated ratio of 85% tumour cells in the dissected tissue uniformly carrying a heterozygous mutation. Further analyses with pure heterozygous samples, such as control cell lines, could be useful to calculate the standard error of this type of measurement for future experiments.

## Discussion

The development of novel targeted therapies has created the need for molecular characterisation of cancers to allow more appropriate treatment decisions. We explored two approaches based on HRM and next-generation sequencing technologies to assess the mutational status of *KRAS*, *BRAF *and *EGFR *in diagnostic settings.

The HRM assay described herein successfully identified hotspot mutations of these genes in 120 FFPE diagnostic specimens. In CRC samples, the mutation frequencies of *KRAS *codon 12/13 and *BRAF *V600E were 44.3% and 13.0%, respectively, and their mutual exclusiveness was confirmed. Our results are coincident with those reported previously [30,31]. In lung cancer, *EGFR *mutations were detected in 11.1% of samples, none of which was mutated for *KRAS*. Despite the small size and heterogeneity of our sample, this percentage is close to the 16.6% reported in a large-scale screening for *EGFR *mutations in Spanish lung cancer patients [5].

Compared to a commercially available mutation test, such as the DxS TheraScreen *KRAS *mutation kit (Qiagen), our HRM assay detected all *KRAS *mutations previously found. Moreover, our HRM *KRAS *mutation analysis has the advantage that it can be performed together with detection of *BRAF *and *EGFR *mutations in the same assay.

As FFPE tissues are the most common clinical specimens available for mutation analysis, assessment of the analytical sensitivity of the *KRAS *HRM assay was performed with patient FFPE-derived DNA instead of control cell lines bearing known *KRAS *mutations. Using dilution series, the assay was able to detect 2.5% mutant alleles or 5% tumour cells carrying a heterozygous mutation. This analytical sensitivity is within the 1.5-6% range obtained with cell lines in other *KRAS *assays based on standard PCR following HRM [30,32,33], and much higher than that of direct sequencing, which continues to be regarded as the "gold standard" although it requires a mutant allele threshold of 10% [34].

Unlike most published HRM studies, which identify wild type and mutant samples according to the normalised temperature-shifted differential plot, we based our analysis on the interpretation of melting peaks. We found this curve to be more sensitive and reproducible, as small variations between samples caused a spread of wild type curves around the baseline of the differential plot. Instead of being a drawback, the presence of multiple melting domains generates a particular curve morphology that differentiates mutant and wild type melting patterns more clearly, even with low amounts of mutant DNA, as seen in the *KRAS *assay.

Another strength of our assay is the use of a touchdown PCR covering a range of annealing temperatures (between 62°C and 54°C) to ensure that all the primer pairs hybridise specifically to the template DNA and adequate amounts of PCR product are finally obtained. Then, the use of a wide melting interval (72°C to 92°C) allows all the amplicons to melt, so they can be analysed together in a single plate, saving time and cost. Optimisation of the reaction volume to 10 μl instead of 20 μl could also be carried out.

The main limitation of our HRM assay is the need to sequence a few positive samples to identify the specific nucleotide alterations. When many types of mutations may exist within an amplicon, as in the case of *KRAS *exon 2, it is difficult to assign a melting profile to each one, even more so if, as we observed, the pattern varies with the amount of the mutant allele. Moreover, clinically important mutations and neutral variants generate almost identical curves and can be confused, as occurs with L858R and P848L mutations in *EGFR *codon 21 (not shown). Thus, direct Sanger sequencing is always necessary to avoid misdiagnosis. However, in a lower mutant allele concentration, Sanger sequencing may be insufficient to validate the results (Figure 2B), and a much higher sensitivity and accuracy may be necessary in some of the scenarios discussed above. We achieved our result with ultra-deep pyrosequencing of *KRAS *amplicons with GS Junior 454, which determined the frequencies of mutant alleles and confirmed the known *KRAS *genotypes. This sequencing experiment also demonstrated excellent performance in terms of throughput and reads per run. The five *KRAS *amplicons were similarly represented, with 9 to 12 × 10^3 ^reads per sample, an excess with respect to the depth of coverage required for accurate assessment of somatic mutations, considering that a 1% variation of a single-base change and multibase deletion will need 5,000-fold coverage to obtain a good statistical chance of 50 variation reads [35]. Moreover, the use of MIDs allowed massively parallel sequence analysis of multiple samples to be performed.

## Conclusions

In conclusion, our HRM assay is a simple, robust and inexpensive method that allows multiple mutation hotspots to be rapidly screened and is thus highly suited to mutation detection in DNA derived from FFPE tissues. Ultra-deep pyrosequencing of *KRAS *amplicons with GS Junior 454 proved to be a highly sensitive and quantitative technique to analyse somatic mutations in cancer specimens, and which can also be used in a high-throughput assay.

## Competing interests

The authors declare that they have no competing interests.

## Authors' contributions

EB designed the study, carried out DNA extraction, designed and tested the primers, performed HRM and pyrosequencing studies, and drafted the manuscript. IJ obtained the FFPE sections from tumour samples and participated in the design of the study. IH participated in HRM and pyrosequencing studies. MJG, MD and IM participated in HRM assays. BM carried out DNA extraction. AA participated in the design and coordination of the study. JAGA and MB participated in the validation of the results. MC conceived the study, participated in its design and coordination, and helped to draft the manuscript. All authors have read and approved the final manuscript.

## Pre-publication history

The pre-publication history for this paper can be accessed here:

http://www.biomedcentral.com/1471-2407/11/406/prepub
